# Male Partners of Infertile Couples with Seminal Infections of Human Papillomavirus Have Impaired Fertility Parameters

**DOI:** 10.1155/2017/4684629

**Published:** 2017-08-01

**Authors:** Edilson Damke, Fábio A. Kurscheidt, Valério A. Balani, Karen I. Takeda, Mary M. T. Irie, Fabrícia Gimenes, Marcia E. L. Consolaro

**Affiliations:** ^1^Clinical Cytology and STD Laboratory, Department of Clinical Analysis and Biomedicine, State University of Maringá, Maringá, PR, Brazil; ^2^Sperm Analysis Section, São Camilo Laboratory, Maringá, PR, Brazil

## Abstract

Several studies have addressed the impact of viral infections on male infertility. However, it is still unknown whether human papillomavirus (HPV) can alter seminal parameters. The aim of this study was to determine the prevalence of HPV in the semen of male partners of couples seeking fertility evaluation. Additionally, we assessed the possibility that HPV infections affect seminal parameters. A total of 229 semen samples were collected from men in the Sperm Analysis Section of São Camilo Laboratory of Maringá, Brazil, between October 2015 and March 2016. Basic seminal parameters were analyzed, and HPV was detected and genotyped by polymerase chain reaction. HPV DNA was detected in 16.6% of samples. Of these, 10.5% had single type HPV infections, 6.1% had multiple HPV infections, 5.7% had exclusively high-risk HPV, and 6.1% had exclusively low-risk HPV. Samples positive for single and multiple types of HPV were associated with abnormal viscosity, and samples positive for multiple HPV types were also associated with hypospermia, higher pH, and increased leukocyte numbers. These findings suggest that the male partners of infertile couples with seminal HPV infections may have prostate disturbances indicative of glandular dysfunction, which may influence fertility.

## 1. Introduction

Human papillomavirus (HPV) is one of the most common viral sexually transmitted diseases in both males and females worldwide [1]. Low-risk types of HPV (LR HPV) can cause genital warts, whereas persistent infection with high-risk types of HPV (HR HPV) is associated with anogenital and oropharyngeal cancers. In males infected with HPV, the virus can be found not only in the anal region, perineum, scrotum, glans, penile shaft, and urethra [2, 3] but also in the reproductive system (testis, epididymis, and ductus deferens) [4, 5]. Moreover, the presence of HPV in semen has been documented previously [6–8]. However, it is still unknown whether HPV infection can affect semen fertility parameters.

Once it became established that the virus is primarily transmitted through direct epithelial contact [8], little attention was paid to the presence of HPV in semen. However, HPV DNA was detected in 10% of semen samples from asymptomatic, sexually active young adult men [5], and Kaspersen et al. [8] found that 16.0% of semen from sperm donors contained HPV. HR HPV and multiple HPV infections were detected in 61.9% and 5.3% of these individuals, respectively. Additionally, HPV infection of the penile epithelium was found to be associated with the presence of HPV in semen, suggesting that seminal HPV might result from exfoliation of HPV-infected penile keratinocytes [9]. As a result, semen samples have high diagnostic value for the assessment of HPV infection in asymptomatic males [10].

So far, conflicting results have been reported on the effects of HPV infection on semen quality [9, 11–17]. Most previous studies on this topic were performed on relatively small cohorts [16–18]. Expanding knowledge in this area could provide valuable insight for guidelines on whether HPV testing is necessary in the diagnostic work-up for subfertile couples and in assisted reproduction procedures [9, 17]. The aim of this study was to determine the prevalence of HPV in the semen of randomized male partners of couples seeking fertility evaluation. In addition, the possibility that HPV infection could affect seminal parameters, and thus fertility, was assessed.

## 2. Materials and Methods

### 2.1. Study Design and Approval

**Figure 1:**
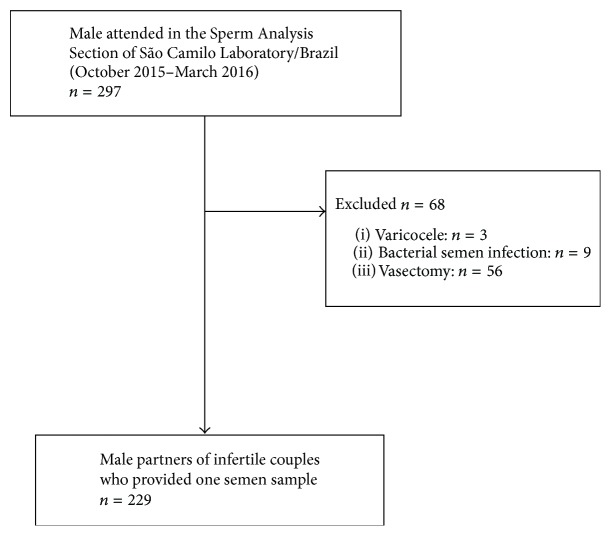
Flow chart of the study.

From October 2015 until March 2016, 297 males were attended in the Sperm Analysis Section of São Camilo Laboratory, Maringá (Paraná, Brazil). Eligible men were 18 years or older and had semen analysis requested by their physician as part of a fertility evaluation, after failing to conceive with their partner after one year of unprotected intercourse. Participants who met the following criteria were excluded: symptoms of genitourinary infections, antibiotic treatment within the previous three months, reproductive system abnormalities (e.g., a varicocele), a history of vasectomy, and infertility therapy in the preceding year. Additionally, subjects who tested positive for* Chlamydia trachomatis*,* Ureaplasma urealyticum*,* Mycoplasma hominis*,* Mycoplasma genitalium,* and* Neisseria gonorrhoeae* by multiplex polymerase chain reaction assay performed by us [19] were excluded (Figure 1). Participation was voluntary and participants were properly informed about the aim of the study. At recruitment, written informed consent was obtained from all study subjects, who were enrolled only after signing the informed consent form. The study was approved by the Committee for Ethics in Research Involving Humans at the State University of Maringá (UEM)/Paraná, Brazil (permission #1163409/2015).

### 2.2. Laboratory Methods

#### 2.2.1. Semen Samples

Prior to semen collection for analysis, men were asked to abstain from sexual intercourse or masturbation for 3–5 days. Before semen collection, subjects were instructed to wash their hands with soap, and all samples were collected after urinating and washing the glans penis and coronal sulcus with soap and water. Semen was collected by masturbation and ejaculation directly into standard sterile containers that had previously been shown not to have any cytotoxic effects on human spermatozoa. Participants avoided contact with the interior wall of the sterile container to prevent sample contamination. Freshly collected semen was immediately incubated at 37°C for 15–60 min for liquefaction. Afterwards, samples were homogenized and 300 *μ*L was transferred to tubes containing 1.0 mL of sterile 0.9% NaCl solution and immediately stored at −80°C until genomic DNA could be extracted.

#### 2.2.2. Semen Analysis

Detection of basic seminal parameters was performed according to criteria published by the World Health Organization [20]. The following variables were measured: seminal volume, pH, viscosity, sperm concentration, vitality, progressive motility (category [a + b]), morphology (normal forms), leukocytospermia, and hematospermia. Lower reference limits for normal semen characteristics are ≥7.2 for pH of semen; 1.5 mL for seminal volume; 15 × 10^6^/mL for sperm concentration; 32% for progressive motility (PR, %); 58% for sperm vitality (live spermatozoa, %); 4% for sperm morphology (normal forms, %); and <1.0 for leukocytes. The terms used to describe samples with values lying outside the reference range were hypospermia for seminal volume; oligozoospermia for sperm concentration; asthenozoospermia for progressive motility; necrozoospermia for sperm vitality; teratozoospermia for sperm morphology; and leukocytospermia [20].

#### 2.2.3. HPV Detection and Genotyping

To remove any polymerase chain reaction (PCR) inhibitors from the semen, samples were incubated for 15 min with proteinase K in phosphate buffered saline and then centrifuged. DNA was extracted using an AxyPrep™ Body Fluid Viral DNA/RNA Miniprep Kit (Axygen, CA, USA) according to the manufacturer's instructions. The quality and quantity of purified DNA were determined by spectrophotometry on a NanoDrop 2000 Spectrophotometer (Thermo Scientific, Wilmington, USA). HPV was detected by PCR using the primers MY09 and MY11 [21]. This reaction produced a final amplified product of 450 base pairs (bp). The amplification reaction was performed as described by Gimenes et al. [19]. The quality of the DNA was tested by amplification of a 268 bp gene fragment of the human *β*-globin gene using the primers GH20 and PC04. Two types of controls were used in the reaction: a sample without DNA (negative control) and an HPV-positive sample (positive control). Final amplified products were loaded onto a 1.0% agarose gel stained with 150 ng/*μ*L ethidium bromide and subjected to electrophoresis in a horizontal tank at 110 V for 45 min in 0.5x TBE buffer (45 mM Tris-borate, 1 mM EDTA, pH 8.0). A 100 bp marker (Invitrogen, Carlsbad, CA, USA) was used as a size standard. The amplified DNA fragments were visualized on a transilluminator with UV light and then photographed. HPV was genotyped by PCR-Restriction Fragment Length Polymorphism (PCR-RFLP) analysis, in which amplified DNA was cleaved with restriction enzymes to generate DNA fragments of different molecular sizes. Aliquots of each amplified product were subjected to digestion with the restriction enzyme HpyCH4V (New England Biolabs, Ipswich, MA, USA) as described by Santiago et al. [22]. To better differentiate among HPV genotypes that have similar RFLP patterns, such as HPV 11/30, 18/68, 44/55, and 61/83/84, a second enzyme, NlaIII (New England Biolabs, Ipswich, MA, USA), was used as described by Chen et al. [23]. The restriction digest fragments were subjected to electrophoretic analysis on 8% polyacrylamide gels. Both 100 and 25 bp ladders (Invitrogen, Carlsbad, CA, USA) were used as molecular size standards. After electrophoresis, polyacrylamide gels were analyzed using LabImage ID software (Loccus Biotechnology, Cotia, SP, Brazil), and the size of each fragment was determined. Genotyping was performed by comparing the molecular weights of fragments for each HPV genotype as described by Santiago et al. [22]. A total of 39 individual HPV genotypes can be determined by the PCR-RFLP method: 17 genotypes are considered to be either HR or potentially HR (16, 18, 31, 33, 35, 39, 45, 51, 52, 53, 56, 58, 59, 66, 68, 73, and 82); 22 LR genotypes are not associated with carcinogenesis (6, 11, 30, 34, 40, 42, 43, 44, 54, 55, 61, 62, 64, 67, 69, 70, 72, 74, 81, 83, 84, and 91); and the carcinogenic risk for one genotype has not yet been determined (26) [22–25].

#### 2.2.4. Statistical Analysis

Seminal parameters were compared between HPV-positive and HPV-negative groups, HR HPV-positive and HR HPV-negative groups, LR HPV-positive and LR HPV-negative groups, and multiply infected HPV-positive and multiply infected HPV-negative groups using Chi-square tests. The means of age and seminal parameters were compared in the total study population and in strata of HPV presence as follows: HPV-negative; mutually exclusive HPV-positive subgroups (HPV DNA-positive, exclusively HR HPV-positive, exclusively LR HPV-positive, and HPV-positive for multiple infections) by one-way analysis of variance (ANOVA). Two-sided *p* values <0.05 were considered statistically significant. Statistical analysis was performed using GraphPad Prism 6.0 (San Diego, CA, USA).

## 3. Results

After written consent, a total of 229 participants who passed the inclusion and exclusion criteria of the study provided one semen sample each (Figure 1). Participants' mean age was 32.87 ± 6.6 years (range 18–52).

The HPV infection rates in semen samples are summarized in Table 1. Overall, 16.6% (38/229) of the total study population was HPV-positive. Single HPV infections occurred in 10.5% (24/229) of the samples and in 63.2% (24/38) of HPV-positive semen samples. Infections with multiple types of HPV occurred in 6.1% (14/229) of the samples and in 36.8% (14/38) of HPV-positive semen samples. In 64.3% (9/14) of samples with multiple HPV types, both LR and HR HPV types were detected simultaneously. HR HPV was detected in the majority of semen samples positive for HPV multiple infections (13/14; 93.0%). HR HPV types were exclusively present in 5.7% (13/229) of samples in single or multiple infections, while 6.1% (14/229) of the samples contained LR HPV types exclusively. The genotype distribution is shown in Table 2. HR HPV16 was the most prevalent genotype in single and multiple infected samples (9/38; 23.7%), followed by LR HPV61 (6/38; 15.8%), HR HPV82 and LR HPV43 (5/38; 13.2% each), and HR HPV58 and LR HPV72 (4/38; 10.5% each).

Table 3 displays the correlation between HPV-positive semen samples and adverse effects on seminal parameters. HPV DNA-positive samples were strongly associated with abnormal seminal viscosity (*p* = 0.0005), and samples with multiple HPV infections were associated with hypospermia (*p* = 0.01) and abnormal seminal viscosity (*p* = 0.0002). Interestingly, semen samples negative for multiple HPV infections were associated with normal seminal viscosity (*p* = 0.03). Still, samples that were HR HPV-positive had a statistically borderline association with abnormal seminal viscosity (*p* = 0.057).

Table 4 lists the means of participant ages and seminal parameters in the total study population and in strata by HPV presence (HPV-negative samples and mutually exclusive HPV-subgroups). The presence of HPV, HR HPV, or LR HPV (separately or in combination) in semen was not related to the mean age of the participants. Having multiple HPV infections was associated with a higher mean seminal pH and a higher mean number of leukocytes (*p* = 0.0003 and *p* < 0.0001, resp.).

## 4. Discussion

To our knowledge, this is the first study in Brazil and Latin America to detect and genotype HPV in a large cohort of male partners of couples seeking fertility evaluation and address the possibility that HPV infection may affect seminal parameters. The results of this study reveal that HPV infection in semen is rather common in male partners of infertile couples. Furthermore, we demonstrated an association between HPV seminal infections, particularly infections involving multiple HPV types, and reduction in seminal volume, abnormal viscosity, and elevation of seminal pH. These changes could potentially play important roles in male subfertility and/or infertility.

The possibility that HPV infection could affect seminal parameters and thus fertility is a highly debated topic in the field of human reproduction [2, 4, 26]. We detected an overall HPV prevalence of 16.6% of the male partners of couples seeking fertility evaluation, which is in accordance with a recent meta-analysis [10] describing a pooled prevalence of 16% in fertility clinic attendees and with a recent study [9] showing an HPV prevalence of 14.9% in the male partners of infertile couples. Other studies in asymptomatic men have reported seminal HPV prevalence ranging from 2 to 38.1% [9, 10, 14, 17], with 16–26% of semen donors [8, 27] and 3–36% of fertility clinic attendees testing positive for HPV [12, 13, 15].

HPV16 was the most prevalent genotype detected in semen samples infected with both single and multiple types, which is in accordance with other recent studies [9, 10]. However, other usually frequent types, such as HPV6, HPV11, and HPV45, were not detected in this study. In other recent studies, HPV51, HPV52 [27], HPV45, HPV52, HPV18/59 [15], HPV53, and HPVCP61 [28] were the most prevalent genotypes in semen. These data suggest that the prevalence of HPV genotypes may vary based on geographic area. Additional factors such as sociodemographic characteristics, lifestyle, and sexual behavior of the male participants likely contributed to the differences in HPV genotype prevalence. However, as our study was not based on medical records, we do not have data regarding sociodemographic characteristics of our participants.

The high prevalence of HPV DNA and more specifically HPV16 in the semen of asymptomatic males in our study supports previous evidence that semen functions as a transport medium for HPV from the male genital tract to the female cervix/uterus [9] and highlights concerns that men with semen infected by HR HPV can potentially be HPV transmitters for a long time. A recent study reported that HPV DNA can be found in semen for 15.3 months and that in males, oncogenic genital HPV infections are more likely to persist for six months or more than nononcogenic infections (61% and 27%, resp.) [29].

Male fertility depends on the two equally important major components of semen: healthy spermatozoa (in terms of their vitality, motility, and morphology) and the composition of seminal fluid, which is important for sperm function. There was no significant association between HPV infection and sperm concentration, motility, or vitality, as recently described by Luttmer et al. [9]. However, our observations that HPV infection can be associated with reduced semen volume, abnormal viscosity, and increased pH deserve some consideration.

Secretory products from the seminal vesicles and prostate are crucial for sperm motility, viability, and chromatin stability, but they are also important for semen coagulation and liquefaction. Seminal vesicles secrete the major fraction of the ejaculate (≈60%) [20]. They produce compounds that play significant roles in seminal physiology, such as fructose, semenogelin-I (the predominant component of the coagulum), and sperm motility stimulators [30–32]. Seminal hyperviscosity suggests deficient secretory activity of the seminal vesicles [33]. Although volume and pH have less clinical value, they can also yield significant information. The prostate secretes 30–35% of the ejaculate [20] and produces several compounds that are available for analysis in the seminal plasma, including enzymes for semen liquefaction. Reduced levels of prostate markers are indicative of glandular dysfunction that often associates with abnormalities in pH (≥7.8), volume (decreased or increased), liquefaction, and/or viscosity. Our data revealed significant associations between HPV-positive semen samples and hypospermia, abnormal seminal viscosity, higher mean pH, and higher mean number of leukocytes, particularly in semen samples positive for HPV multiple infections. Together, these results indicate that HPV-positive infections in the semen of male partners of couples seeking fertility evaluation may have altered proportions of fluid secreted by the major sexual accessory organs, the prostate, and the seminal vesicles. More specifically, HPV seminal infections appear to lead to changes in prostate markers that are indicative of glandular dysfunction, which may influence fertility. In this context, the association between leukocytospermia and the impairment of prostate markers is remarkable, because it can single out the infection site [34]. Furthermore, as most seminal HPV infections are asymptomatic, leukocytospermia may also be an essential diagnostic probe [35].

Limitations of our study include the absence of data on mixed antiglobulin reaction testing, which was not tested in each primary semen analysis but performed only upon physician's request. Secondly, as our study did not include analysis of medical records, it does not include data on the female partner's characteristics, such as age, hormone levels, tubes, pelvic status, and additional male partner characteristics such as clinical analysis of the prostate and seminal vesicles. Still, semen results were taken from only one semen analysis per subject. Finally, our study did not include normal fertile subjects (control group). However, we believe that our results may encourage further studies to evaluate the influence of HPV infection on changes in seminal parameters.

## 5. Conclusions

This study is the first to reveal a significant association between HPV-positive semen and hypospermia, abnormal seminal viscosity, higher mean pH, and higher mean numbers of leukocytes, particularly in semen samples positive for multiple HPV infections. Our data support a model where seminal HPV infections lead to changes in prostate markers that are indicative of glandular dysfunction and could change the proportion of fluids secreted from the prostate and the seminal vesicles. Thus, HPV seminal infections could play an important role in male infertility. These results deserve further attention and additional studies should be conducted with larger cohorts.

## Figures and Tables

**Table 1:** Presence of HPV in total semen samples (*n* = 229).

HPV presence in semen	*n*	Percentage (%)
HPV DNA	38/229	16.6
HPV single infection	24/229	10.5
HPV multiple infection	14/229	6.1
Exclusively high-risk HPV genotype(s)	13/229	5.7
Exclusively low-risk HPV genotype(s)	14/229	6.1

Percentage not sum to total due to rounding; HPV: human papillomavirus; DNA: deoxyribonucleic acid.

**Table 2:** Detected HPV genotypes in semen (both single and multiple infections).

	HPV genotypes	*n*	Percentage (%)
High-risk	16	9	23.7
	82	5	13.2
	58	4	10.5
	53	2	5.3
	56	2	5.3
	66	2	5.3
	18	1	2.6
	31	1	2.6

Low-risk	61	6	15.8
	43	5	13.2
	72	4	10.5
	54	2	5.3
	62	2	5.3
	84	2	5.3
	44	1	2.6
	69	1	2.6
	81	1	2.6
	83	1	2.6

Total HPV-positive samples, *n* = 38; indicated frequencies include presence of types both in single and multiple infections.

**Table 3:** Comparison of semen parameters in HPV-positive semen samples.

	HPV DNA+ (*n* = 38) *n* (%)	HPV DNA− (*n* = 191) *n* (%)	*p*	HR HPV+ (*n* = 13) *n* (%)	HR PV− (*n* = 216) *n* (%)	*p*	LR HPV+ (*n* = 14) *n* (%)	LR HPV− (*n* = 215) *n* (%)	*p*	HPV MI+ (*n* = 14) *n* (%)	HPV MI− (*n* = 215) *n* (%)	*p*
Hypospermia	6 (15.8)	15 (7.8)	0.2	4 (25)	17 (8)	0.3	0 (0)	21 (9.7)	0.6	5 (35.7)	16 (7.44)	**0.01**
Abnormal coagulation time	0 (0)	5 (2.6)	1	1 (8.3)	2 (0.9)	0.1	0 (0)	5 (2.3)	1	0 (0)	5 (2.3)	1
Abnormal viscosity	17 (44.7)	23 (12.4)	**0.0005**	7 (58.3)	33 (15.2)	**0.057**	3 (21.4)	34 (15.8)	0.7	11 (78.5)	29 (13.5)	**0.0002**
Abnormal pH	1 (2.63)	2 (1)	0.4	1 (8.3)	2 (0.9)	0.1	0 (0)	3 (1.4)	1	0 (0)	3 (1.4)	1
Asthenozoospermia	16 (42)	64 (33)	0.5	2 (16.6)	78 (36)	0.5	6 (42.8)	74 (34)	0.6	7 (50)	73 (34)	0.44
Oligozoospermia	10 (26.3)	52 (27)	1	2 (16)	60 (27.6)	0.38	4 (28.5)	58 (27)	1	3 (21.4)	59 (27.4)	1
Necrozoospermia	21 (55)	77 (40)	0.3	4 (33)	92 (42)	0.4	8 (57.1)	88 (41)	0.5	9 (64.2)	87 (40.4)	0.34
Teratozoospermia	6 (15.8)	28 (14.65)	0.8	1 (6.2)	33 (15.5)	0.7	3 (21.4)	31 (14)	0.4	0 (0)	34 (15.8)	0.2
Leukocytospermia	2 (5.26)	10 (5.23)	1	1 (8.3)	11 (5)	0.5	0 (0)	12 (5.6)	1	2 (14.2)	10 (4.65)	0.2
Hematospermia	1 (2.63)	3 (1.57)	0.5	0 (0)	4 (1.9)	1	0 (0)	4 (1.8)	1	1 (7.14)	3 (1.4)	0.2

HPV: human papillomavirus; DNA: deoxyribonucleic acid; +: positive; −: negative; HR: high-risk; LR: low-risk; MI: multiple infection; presented data are not means; *p* values were obtained by Chi-square test; *p* values < 0.05 were considered statistically significant.

**Table 4:** Mean participant age and seminal parameters in the total study population (*n* = 229) and in strata by HPV presence.

	Total group (*n* = 229)	HPV DNA+ (*n* = 38; 16.6%)	Exclusively HR HPV+ (*n* = 13; 5.7%)	Exclusively LR HPV+ (*n* = 14; 6.1%)	HPV MI+ (*n* = 1; 6.1%)	HPV DNA− (*n* = 191; 83.4%)	*p*
Age of participants (years)	34.4 (32–33.7)	33.5 (30.9–36.2)	34.6 (30.7–38.6)	34.3 (30.6–38.15)	34.7 (30.7–37.8)	32.8 (31.9–33.7)	0.9
Volume of semen (ml/ejaculate)	2.9 (2.7–3.5)	2.9 (2.3–3.3)	3.1 (2.2–4.1)	2.5 (1.8–3.1)	2.4 (1.3–3.4)	3.5 (3.3–3.7)	0.85
Coagulation time (minutes)	24.9 (22.7–25.4)	25.1 (24.5–25.7)	24.8 (23.3–26.4)	25.1 (24.2–25.9)	24.2 (18.3–30.1)	25.3 (25.2–25.5)	0.9
pH of semen	7.8 (7.6–7.9)	7.8 (7.7–7.9)	7.8 (7.6–8.0)	7.7 (7.6–7.8)	8.1 (7.7–8.3)	7.7 (7.64–7.71)	**0.0003**
Progressive motility (a + b) (%)	43.6 (40.5–46.6)	42.4 (34.9–49.9)	48.6 (37.5–59.8)	42.2 (27.3–57.2)	35 (18.4–51.4)	49.8 (40.5–54.4)	0.7
Sperm count (×10^6^/ml)	47.2 (41–58)	46 (31–61)	53 (24–81)	50 (22–79)	39 (6–71)	48 (21–72)	0.7
Vitality (%)	54.1 (49.7–61.9)	54.5 (46.1–63.1)	62.3 (52.5–72.2)	54.4 (37.5–71.3)	39.9 (26.8–56)	59.4 (55.9–63)	0.2
Leukocytes (×10^3^/ml)	322.7 (309–346)	252.6 (207–298)	229.2 (143–315)	336.7 (275–398)	485.7 (31.59–939.8)	345 (323.7–366.3)	**<0.0001**

HPV: human papillomavirus; DNA: deoxyribonucleic acid; +: positive; −: negative; HR: high risk; LR: low risk; MI: multiple infection; presented data are means (95% CI); CI: confidence interval; *p* value was obtained by one-way ANOVA, comparing the HPV-negative group with the four mutually HPV exclusive subgroups; *p* values < 0.05 were considered statistically significant.
